# Active Patient Involvement in Healthcare Education in the Global South: Perspectives From Pakistan

**DOI:** 10.1111/tct.70184

**Published:** 2025-08-26

**Authors:** Ayesha Jawwad, Adeena Sajid, Subha Ramani, Herman Popeijus, Marjan Govaerts

**Affiliations:** ^1^ School of Medicine Ulster University, United Kingdom & School of Health Professions Education, Maastricht University the Netherlands; ^2^ Shifa College of Dentistry Islamabad Pakistan; ^3^ Department of Medicine and Brigham Education Institute Brigham and Women's Hospital and Harvard Medical School Boston Massachusetts USA; ^4^ Department of Nutrition and Movement Sciences Institute of Nutrition and Translational Research in Metabolism the Netherlands; ^5^ Faculty of Health, Medicine and Life Sciences Maastricht University the Netherlands; ^6^ School of Health Professions Education Maastricht University the Netherlands

**Keywords:** cultural competence, global south, health professions education, patient participation, qualitative research

## Abstract

**Introduction:**

Direct and active patient involvement in health professions education (HPE) can improve learners' clinical skills and preparedness for patient‐centred care. Most research on active patient involvement is from global north (GN) regions where cultural contexts differ from those in the global south (GS). In most GS contexts, patients are not actively engaged in educational activities. Understanding how patients in these regions perceive their potential roles in education is essential to improving HPE not only in the GS but also in culturally diverse GN settings.

**Methods:**

This qualitative study explored patient perspectives on active involvement in HPE using a theory‐informed inductive approach. We conducted semistructured interviews with 13 patients at a dental teaching hospital in Pakistan. Data were analysed using Braun and Clarke's thematic analysis. Iterative team discussions were held to reach consensus during coding and theme development. Hofstede's cultural dimensions theory was used as a lens to guide conceptualization and mapping of final themes.

**Results:**

Six key themes, rooted in cultural values, captured patients' views on active participation in education: contribution to societal good; spiritual and moral obligations; nurturing and supporting students; self‐doubts; fear of negative consequences; and balancing responsibilities.

**Conclusion:**

Patient perceptions shaped by cultural factors can act as facilitators or barriers to their participation in HPE. Educators should engage with diverse communities and use culturally sensitive approaches to support patient participation. In a globally interconnected educational landscape, understanding and respecting cultural differences when involving patients in education can enable educators to better prepare future healthcare professionals.

## 1 Introduction

Patients' active involvement in education has emerged as a transformative force in healthcare. Active involvement refers to patients taking on various educational roles such as teachers, assessors, evaluators, mentors and contributors to curriculum design [1]. Evidence suggests that such active patient participation not only enhances the educational experiences of future healthcare professionals but also increases patients' understanding of healthcare systems [2, 3]. It improves students' preparedness for patient‐centred care, strengthens their communication skills and fosters empathy [4, 5]. Moreover, exposure to diverse patient perspectives enriches students' clinical practice [6]. From a patient perspective, recognition as active partners in education can empower them and improve their health outcomes [7]. However, much of the existing research on active patient involvement is currently limited to regions of the world known as the global north (GN) [8], which have specific cultural values or dimensions that shape their educational landscapes.

Culture is a complex and dynamic concept, encompassing shared beliefs, values, customs and behaviours within a society [9]. It is often described as ‘fuzzy’ due to its broad and variable nature, influencing nearly all aspects of human interaction, including education [10]. In health professions education (HPE), culture influences teaching methods, student–teacher dynamics and expectations for learning outcomes. Research shows that teaching strategies often require adaptation when applied in diverse global contexts [11]. Thus, educational practices developed in GN settings may need to be realigned to match cultural nuances shaping educational interactions in other regions of the world.

In GN countries, educational approaches usually emphasize autonomy, critical thinking and collaborative learning through student–teacher partnerships [12]. In contrast, many global south (GS) regions [8] have different historical, sociopolitical and economic conditions that influence educational practices and outcomes [13]. In GS settings, educational structures are typically more hierarchical, emphasize deference to authority and prioritize collective well‐being over individual expression [14]. Understanding cultural differences is important as they could shape the beliefs, assumptions and behaviours of students, educators and patients. This knowledge is essential for guiding effective approaches to active patient involvement in education [15].

While many theories on culture have been developed, Hofstede's cultural dimension theory specifically and clearly identifies different dimensions of culture (Table 1) that influence social behaviours and interactions. Understanding these different aspects is key for working across cultures or in culturally diverse environments like educational and healthcare settings [16].

**TABLE 1:** Dimensions of culture adapted from the Hofstede's Model [16].

Power distance	The degree to which a society accepts unequal distribution of power; higher power distance reflects more hierarchical relationships and deference to authority.
Individualism vs. collectivism	Indicates whether people see themselves as independent individuals or part of a group; individualist cultures value autonomy, while collectivist cultures emphasize group harmony and shared responsibility.
Masculinity vs. femininity	Reflects cultural preferences for competitiveness and achievement (masculinity) versus cooperation, empathy and quality of life (femininity). Rigid gender roles persist in masculine societies
Uncertainty avoidance	The extent to which a culture feels uncomfortable with ambiguity; high uncertainty avoidance favours clear rules, structure and predictability.
Long‐term vs. short‐term orientation	Describes whether a culture emphasizes long‐term goals and persistence or favours tradition, stability and short‐term outcomes based on the choice of focus for people's efforts: the future or the present and past
Indulgence vs. restraint	The extent to which societies permit the free expression of desires and enjoyment versus valuing restraint, discipline and adherence to social norms

When patients participate in HPE, their cultural backgrounds likely influence their perceptions of their roles, interactions with students and overall contributions to the educational process [17]. This is supported by findings in healthcare settings where culture influences doctor–patient relationships and communications, for instance, during consultations and in shared decision‐making [18]. Cultural factors shape how patients express concerns, preferences and expectations in verbal and non‐verbal communication [19]. They also shape conversations about sensitive issues, key to building trust and collaboration between patients and healthcare professionals [20]. Understanding cultural influences can ensure that patient involvement in educational settings is meaningful, respectful and bidirectional: gaining an in‐depth understanding of patient perspectives can help educators and policymakers develop culturally sensitive approaches to patient involvement and prepare graduates to serve GS communities more effectively. Additionally, insights from GS contexts are highly beneficial for GN education, allowing for better informed support of increasingly diverse patient populations. The purpose of this study is to explore patient perspectives regarding their active involvement in undergraduate HPE in a specific GS setting.

## 2 Methods

### 2.1 Study Design

We used qualitative research methodology [21] following a theory‐informing inductive approach as outlined by Varpio et al. [22]. This allowed us to explore and integrate relevant theoretical frameworks during data analysis, mapping emerging findings to our chosen framework for interpretation.

### 2.2 Study Setting

This study was conducted at Shifa College of Dentistry and its affiliated hospital in Islamabad, Pakistan. The college offers a 4‐year undergraduate dental programme, with approximately 50 students graduating every year. During the final years of undergraduate training, students perform various clinical dental skills on patients from diverse social backgrounds. Dental consultants closely supervise and teach students. Currently, patients are not actively involved at any stage of the educational process.

### 2.3 Ethical Approval

Ethics approval was obtained from the Shifa Tameer‐e‐Millat University Ethics Board (Approval number: R1‐SCD‐2023/2). All participants provided informed consent prior to their involvement in the study.

### 2.4 Participants and Sampling

We used purposive sampling to select participants who could provide rich information to achieve study objectives [23]. Eligible participants were adult patients who had been treated by dental students within the last 3 months. After 11 interviews, we did not identify new themes relevant to the study aims. We conducted two more interviews (total of 13) to ensure data sufficiency. An overview of participants' characteristics can be found in Table 2.

**TABLE 2:** Demographic details of the study participants.

Participant no.	Age range	Gender	Education level
**1**.	60–65	Male	High school or equivalent
**2**.	20–25	Female	High school or equivalent
**3**.	45–50	Female	Below high school
**4**.	35–40	Female	Uneducated
**5**.	25–30	Female	University education
**6**.	50–55	Female	Uneducated
**7**.	20–25	Female	High school or equivalent
**8**.	50–55	Female	University education
**9**.	50–55	Female	University education
**10**.	60–65	Male	High school or equivalent
**11**.	20–25	Female	High school or equivalent
**12**.	30–35	Male	University education
**13**.	20–25	Female	High school or equivalent

### 2.5 Data Collection

We developed a semistructured interview guide (Data S1), [24] informed by existing literature [2, 25, 26] on patient involvement in education. Questions focused on patients' experiences with dental students, their potential roles in the educational process and their willingness to provide feedback to students. The guide was tested in a pilot interview and revised for clarity. Interviews, lasting 25–40 min, were conducted at the dental hospital or online, with participants choosing their preferred language (Urdu, English or both). All interviews were audio‐recorded, with field notes capturing non‐verbal cues. We conducted member checking by summarizing participant narratives at the end of each interview to confirm the accuracy of our interpretation. Transcriptions were verified and checked for accuracy by two members of the team (AJ and AS) and deidentified for analysis. Insights from concurrent data collection and analysis informed later interviews. As analysis progressed, we focused on Sections 2 and 4 of the interview guide, as participants' narratives emphasized how cultural norms and values shaped their perspectives on involvement in education.

### 2.6 Data Analysis

We used Braun and Clarke's thematic analysis [27] for interpretation of data. All members of the research team independently coded the transcripts from the first three interviews. Codes were then compared and discussed within the research team to refine the coding framework for further data analysis (AJ). Throughout data analysis, interpretation of codes and development of themes took place through iterative team discussions. We used Hofstede's cultural dimensions theory [16] as a theoretical lens during the final stage of analysis to interpret and map key themes that were identified.

### 2.7 Reflexivity

Throughout the research process, we reflected on how our backgrounds and roles could influence data collection and interpretation. AJ had worked previously at the study institution and had knowledge of the clinical and educational context. She engaged in self‐reflection and team discussions to minimize potential biases. AS, a recent graduate of the institution, conducted the interviews. She had no treatment role with interviewees or educational role at the college; thus, she was not in a position of power over participating patients. SR, with experience in both the GS and GN, contributed her clinical and qualitative research expertise, while MG and HP offered viewpoints shaped by their academic work in HPE in the GN. This diversity of skills and experiences enriched our data interpretation and narrative.

## 3 Results

We developed six key themes presented in Table 3. Together, these themes provided insights into patients' beliefs and assumptions about active involvement in education and how these may be influenced by different factors related to their personal and cultural contexts. In the following paragraphs, each theme will be described in detail along with representative quotes.

**TABLE 3:** Key themes and corresponding elements in patient involvement in education.

Theme	Description of key elements under each theme	Participant quotes
**Working for societal good**	Social responsibility, community impact, advocacy for student‐led care	‘This will help a poor person, and this can be a possibility that even if we did not get good treatment may be the next person gets a good treatment’. (patient 7) ‘I feel very good contributing, because these children are also someone's children, if they succeed their success will help in the betterment of society’. (patient 3)
**Spiritual and moral obligations**	Fulfilment of religious duty, prayers for students' success	‘ … .. because prayer goes a long way, they might not be able to see it now, but prayer always comes from the heart and this prayer from the heart always has effects on the future of other people’. (patient 2)
**Nurturing and supporting students**	Supporting students' professional growth, cultural value of guiding the younger generation	‘I would want them to know that they have done well, and they should feel appreciated. This is their right’. (patient 1)
**Self‐doubt**	Self‐doubt, uncertainty about role legitimacy, societal expectations	‘I am not the right person to judge … I can only say thank you very much. Yes, I am feeling comfortable, or I am not feeling comfortable. That is the only thing I can tell …. I do not know what the details are …. that should be checked so that can only be done by the doctor’. (patient 12)
**Fear of consequences**	Fear of repercussions, concern for maintaining patient‐ provider relationships	‘A patient might be scared that this might cause some trouble for him (the patient) in the future, the reality is that people do not like to talk’. (patient 6)
**Balancing responsibilities**	Time constraints, balance of domestic/work responsibilities, especially for women	‘There is an issue of giving time to this you see … it is very difficult to leave the house again and again. If someone is a housewife and she has to look after kids and if someone is a working woman so taking leave from work again and again can cause issues as well’ (patient 9)

### 3.1 Working for Societal Good

Patients saw their involvement in education as giving back to their community. They valued their contributions as a form of social responsibility, emphasizing how their participation could benefit both themselves and society at large. Many believed that aiding in the education of future healthcare professionals was a contribution to a much greater cause.

…. this is a benefit that will give many rewards to the society. I, as a patient cannot do anything else, but I am definitely willing to help you and contribute to society because this is what I can do and this is what we as patients should do. (patient 1)

Some patients expressed a strong emotional investment in students' success, framing their involvement as a shared responsibility. Others saw an active role in promoting student‐led care as a way to improve trust in student treatment and ensure greater access to affordable healthcare within their communities.

### 3.2 Spiritual and Moral Obligations

Some patients believed that prayers and blessings had the power to shape students' careers. For these patients, involvement in education was deeply rooted in religious beliefs and carried significant meaning. They felt their participation fulfilled a religious obligation and viewed it as a moral responsibility. This strong sense of purpose motivated them to actively support the students' learning.

You see religion always tells us to say the right thing and helping these students is the right thing. (patient 11)

### 3.3 Nurturing and Supporting Students

In addition to moral and spiritual support, patients also saw themselves as playing a nurturing role in students' education. They took pride in being able to contribute positively to the students' learning experiences, recognizing that their role could make a tangible difference in the students' training.

I have felt good if somebody (i.e., some student) can get their work done because of me. (patient 7)

For many patients, this role extended beyond passive participation in their treatment relationships; they felt responsible for promoting students' confidence and motivation. Participants indicated that this nurturing attitude aligned with deeply ingrained cultural values, emphasizing the responsibility of guiding and supporting the younger generation.

One should help participate in these things for students … helping those who are younger is part of our culture. (patient 12)

### 3.4 Self‐Doubt

A few patients expressed doubts about their competence to be formally involved in education. The unfamiliarity of being involved in the educational process and providing feedback and their own limited education seemed to leave them feeling uncertain about their role.

I am not educated much so maybe I would not be the right person to say; I am a simple person; I have never done this before, so I feel a little uncomfortable. (patient 2)

These feelings of inadequacy appeared to be tied to their social roles and perceived limitations. Several patients emphasized that while they could comment on their own treatment, they felt unqualified to speak on broader medical or professional issues related to students. Societal expectations appeared to reinforce this hesitation, as some questioned whether patients should even have a role in education, expressing concerns that their input might not be valued or taken seriously.

A patient is a patient … should he even have a role? (patient 6)

### 3.5 Fear of Negative Consequences

The fear of potential negative consequences for themselves as well as others could deter patients from participating in education. Many feared that offering honest feedback could affect their own treatment or create conflicts with healthcare providers. The possibility of being viewed negatively or disrupting clinical routines would lead some to withhold their opinions, contributing to a culture of silence.

Patients also worried that their feedback could unfairly impact a student's future, particularly if the student was having a difficult day. Some even justified instances of inappropriate behaviour, attributing them to the pressures students faced rather than viewing these as issues to be addressed.

I would not want to be blamed if something bad were to happen to the student because of my feedback. I mean, they are working hard and maybe they could be rude because they are having a bad day. (patient 3)

### 3.6 Balancing Responsibilities

Time constraints and daily obligations emerged as major barriers to being actively involved in student education. Many patients found it difficult to allocate time for feedback, particularly when their schedules were already packed with work, personal or family commitments. Some questioned the feasibility of providing input when their priority was receiving treatment and returning to their responsibilities as quickly as possible.

Time can be an issue … like if you ask me for feedback, my treatment might be about to start or I might have to go back for my job, so why should I give all this feedback to when time is short …? (patient 12)

Women, especially those balancing domestic and caregiving duties, expressed additional challenges in taking on an educational role. Household responsibilities often made it difficult to leave home repeatedly, and for working women, taking time off from their jobs was seen as an added burden.

## 4 Discussion

This study contributes to our understanding of patients' perspectives on active participation in HPE within one GS context. Patients viewed their involvement as a social responsibility, driven by cultural and moral beliefs and a desire to support student growth. However, many expressed self‐doubt, fear of repercussions and concerns about societal acceptance of their role in education. Despite these concerns, participation was often linked to a sense of pride and a cultural commitment to guide the younger generation.

*Patients viewed their involvement as a social responsibility, driven by cultural and moral beliefs*.

**FIGURE 1:**
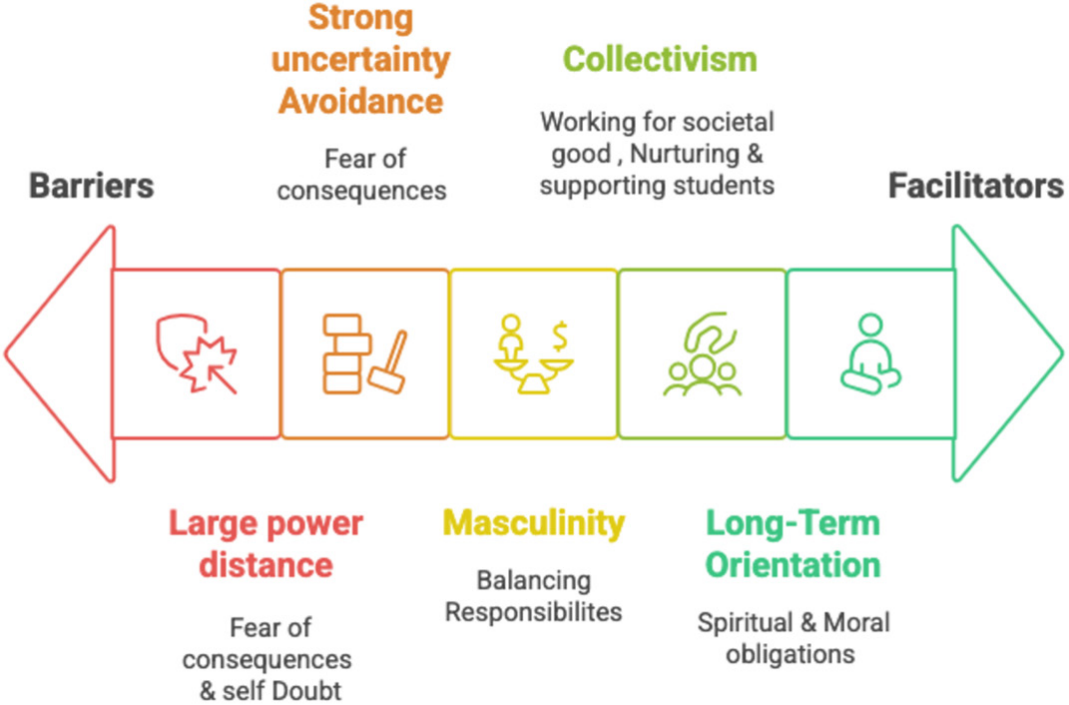
Cultural facilitators and barriers facilitating active patient involvement in HPE in Pakistan mapped onto 5 of Hofstede's cultural dimensions (collectivism, long‐term orientation, power distances, uncertainty avoidance and masculinity).

Hofstede's cultural dimensions theory [16] helped us to conceptualize how cultural values could shape patient participation in education. Of the six dimensions described in Hofstede's framework, we were able to map identified themes to collectivism vs. individualism, power distance, uncertainty avoidance, masculinity vs. femininity and short‐ vs. long‐term orientation. The last, indulgence vs. restraint, was not clearly visible in our data. By mapping our findings onto these dimensions (see Figure 1) we provide some insights into cultural facilitators and barriers that could affect patient involvement in HPE within GS settings similar to the setting of our study. We also compare our results with GN literature to offer a broader perspective on cultural influences across diverse settings.

### 4.1 Cultural Facilitators to Active Patient Participation

Patients in this study appeared to be motivated by a commitment to contribute to future generations and the long‐term benefits for their communities. This aligns with collectivist and long‐term‐oriented cultures, where contributing to societal welfare is highly valued [28]. In contrast, GN studies suggest patient involvement is often driven by personal benefits, such as peer connection or gaining insight into healthcare systems. Some GN patients, particularly those with negative healthcare experiences, use their participation as a means of advocating for change, reflecting a more individualist perspective [28].

Religious and spiritual beliefs may further reinforce participation in the GS, with many patients viewing their role as a moral or religious duty [29]. This is in line with Hofstede's dimension of long‐term orientation, explained as conformation to societal norms and a prioritization of traditional values and moral continuity [30]. In contrast, GN patients may be more likely to frame participation in terms of secular civic responsibility rather than spiritual fulfilment [31].

Many patients in our study saw themselves as mentors or parental figures, reflecting collectivist and long‐term‐oriented values. This relational dynamic between patients and students therefore seems to be another facilitator: Despite high‐power distance in GS societies [32], the cultural expectation to nurture younger generations may promote patients' willingness to be actively involved. In GN contexts, patients often perceive their role as partners rather than mentors and emphasize personal empowerment [33, 34].

### 4.2 Cultural Barriers to Active Patient Participation

Hofstede's model [16] suggests that in collectivist cultures with high‐power distance, such as those in many GS regions, respect for authority and hierarchical traditions shapes education. This reinforces deference in relationships, including those between students and teachers, as well as patients and healthcare providers. In our study, patients often positioned themselves on the lower end of the power dynamic. Many doubted their ability to provide meaningful feedback and feared negative consequences, even in instances of clearly inappropriate student behaviours. They thus avoided challenging authority, prioritizing stability over confrontation. This self‐censorship reflected cultural values in which hierarchical roles are easily accepted and a broader reluctance to risk negative attention within the healthcare system.

*Many doubted their ability to provide meaningful feedback and feared negative consequences*.

In GN contexts, hierarchical dynamics exist but are less rigid [31, 33], indicating a lower power distance. GN patients, who participate in education view themselves as active partners in healthcare, and expect equal treatment, which fosters open communication and challenges to authority [30]. In contrast, GS patients in our study, influenced by collectivist values, deferred to medical professionals, avoiding actions that could disrupt relationships with healthcare providers.

Time constraints were identified to be a significant barrier to active patient involvement, similar to previous research on patients' perceptions in the GN [35]. For female patients, domestic duties like childcare and household tasks were often the main factor, reflecting traditionally gendered roles common in many GS contexts [36]. This aligns with Hofstede's description of masculine societies, where rigid gender roles persist. However, while GN contexts may offer some structural support for public participation, work‐life balances influenced by different societal norms do remain a challenge for patients in these regions as well [31].

This study has a few limitations. Our participants were selected from a single centre and a single discipline, which may limit the transferability of findings to other healthcare settings. According to Hofstede's theory, cultural values in many countries may lean to one side or the other, but this position is dynamic and along a spectrum—rather than strictly located at one pole. Therefore, our small sample size may not have fully captured the diversity of patient perceptions across different regions of the GS. The GS is not culturally homogeneous, and perspectives may vary significantly across Latin America, Africa and Asia. Additionally, our study focused solely on patients, and we did not capture the perspectives of other key stakeholders, such as educators, learners and administrators. Future research should explore active patient involvement in HPE across multiple sites, healthcare specialties and diverse stakeholder groups to provide a more comprehensive understanding of the factors that influence patient participation.

## 5 Future Directions

Our findings highlight the need for culturally adaptive strategies that address both structural and social barriers to patient participation as educators. A key lesson from this study is the importance of empowering patients as capable contributors to education and fostering conversations about their beliefs and assumptions that guide behaviours. Our participant narratives helped us to develop a set of culturally informed practical recommendations to enhance active patient involvement in diverse HPE contexts summarized in Table 4. Key approaches include emphasizing the importance of patients' roles in education to all stakeholders (trainees, educators and patients), supporting patients in providing feedback through appropriate training and structured opportunities and training healthcare professionals in strategies to engage patients as educational partners.

**TABLE 4:** Practical recommendations for enhancing patient involvement in multicultural HPE settings.

Theme	Practical recommendations for HPE educators and institutions
**Working for societal good**	Develop patient recruitment materials emphasizing their contribution to community health and future care quality Organize community forums where patients share their experiences and reinforce the collective impact of their involvement Highlight and publicize success stories linking patient roles to societal benefits, such as at conferences and newsletters
**Spiritual and moral obligations**	Collaborate with local religious and community leaders to respectfully incorporate spiritual motivations into patient engagement Allow time and space for patients to offer prayers or blessings if culturally appropriate Train educators to acknowledge and affirm patients' moral reasons for participation during sessions
**Nurturing and supporting students**	Facilitate patient‐led mentorship opportunities, such as storytelling or sharing lived experiences Train educators and students to explicitly recognize patients' nurturing roles with verbal appreciation Create safe environment for patients to provide positive encouragement to learners Create feedback sessions encouraging patients to motivate students, reinforcing their positive impact on learner confidence
**Self‐doubt**	Provide patients with simple, jargon‐free briefing sessions explaining their unique insights and importance Offer training along with structured feedback templates to guide patients in communicating observations of learner performance Address cultural factors that limit patient roles through awareness campaigns targeting both patients and healthcare staff
**Fear of negative consequences**	Introduce confidential feedback channels (e.g., anonymous surveys or third‐party mediators) to reduce fear of reprisal Communicate clear institutional policies protecting patients from any negative impact related to their educational participation Assure patients that participation in education and access to healthcare are independent of each other Train students and staff on accepting constructive feedback without judgement or retaliation
**Balancing responsibilities**	Offer flexible participation options such as short sessions before or after clinical visits, or virtual feedback opportunities. Provide logistical support, including transport or childcare services, especially targeting women balancing caregiving duties. Allow scheduling input from patients to accommodate their daily commitments and reduce burden.

For clinicians and educators in the GN, insights from our study offer an opportunity to reflect on how cultural factors can shape patient involvement in education. Recognizing that culture is not static but evolves with migration and globalization, and respecting a patient's cultural values may help build trust and encourage participation, especially among patients from traditionally underrepresented groups in healthcare education. Creating culturally safe environments for patient feedback and addressing barriers to perceived legitimacy as educators could enhance meaningful inclusion of patients from various cultural backgrounds. Evaluating the effectiveness of these strategies and their impact on both educational outcomes and healthcare delivery will be essential in shaping future initiatives.

## 6 Conclusion

Cultural contexts significantly shape education overall and active patient involvement in education. Knowledge from the GN cannot be automatically applied to culturally different educational contexts. As there are major differences in social and educational cultures across GN and GS countries, identifying and understanding cultural values can inform the design of more inclusive and contextually responsive educational practices. Educators must recognize and address cultural differences, using contextually responsive educational practices and adopting culturally sensitive strategies aligned with patients' beliefs, motivations and expectations. This provides a safe environment to encourage active patient involvement in education. Such strategies could help prepare future professionals to better serve diverse communities and possibly improve the effectiveness of healthcare overall.

*Cultural contexts significantly shape education overall and active patient involvement in education*.

## Author Contributions

**Ayesha Jawwad:** conceptualization, methodology, software, writing – original draft, writing – review and editing, investigation, formal analysis. **Adeena Sajid:** investigation, methodology. **Subha Ramani:** supervision, methodology, validation, investigation, writing – review and editing, formal analysis. **Herman Popeijus:** supervision, writing – review and editing, investigation, validation, formal analysis. **Marjan Govaerts:** supervision, formal analysis, validation, investigation, writing – review and editing, conceptualization.

## Conflicts of Interest

The authors declare no conflicts interest.

## Supporting information


**Data S1:** tct70184‐sup‐0001‐Supplementary_File.docx. Supporting Information.

## Data Availability

The data that support the findings of this study are available on request from the corresponding author [AJ]. The data are not publicly available due to information that could compromise the privacy of research participants. The participants of this study did not give written consent for their data to be shared publicly; so, due to the sensitive nature of the research, supporting data are not available.

## References

[1] A. Jawwad , Z. Zaidi , S. Ramani , H. E. Popeijus , and M. Govaerts , “Active and Direct Patient Participation in Health Professions Education: A Narrative Overview of Literature From the Global South,” Patient Education and Counseling 135 (2025): 108734, https://linkinghub.elsevier.com/retrieve/pii/S0738399125001016. 40058146 10.1016/j.pec.2025.108734

[2] A. Towle , L. Bainbridge , W. Godolphin , et al., “Active Patient Involvement in the Education of Health Professionals,” Medical Education 44 (2010): 64–74. 20078757 10.1111/j.1365-2923.2009.03530.x

[3] P. Rowland , M. Anderson , A. K. Kumagai , S. McMillan , V. K. Sandhu , and S. Langlois , “Patient Involvement in Health Professionals' Education: A Meta‐Narrative Review,” Advances in Health Sciences Education 24 (2019): 595–617, 10.1007/s10459-018-9857-7. 30306292

[4] S. W. Dijk , E. J. Duijzer , and M. Wienold , “Role of Active Patient Involvement in Undergraduate Medical Education: A Systematic Review,” BMJ Open 10, no. 7 (2020): e037217. 10.1136/bmjopen-2020-037217 PMC7389514 32718925

[5] A. H. Henriksen and C. Ringsted , “Learning From Patients: Students' Perceptions of Patient‐Instructors,” Medical Education 45, no. 9 (2011): 913–919. 21848719 10.1111/j.1365-2923.2011.04041.x

[6] Z. Setna , V. Jha , K. A. M. Boursicot , and T. E. Roberts , “Evaluating the Utility of Workplace‐Based Assessment Tools for Speciality Training,” Best Practice & Research. Clinical Obstetrics & Gynaecology 24, no. 6 (2010): 767–782. 20598644 10.1016/j.bpobgyn.2010.04.003

[7] A. Towle , H. Brown , C. Hofley , R. P. Kerston , H. Lyons , and C. Walsh , “The Expert Patient as Teacher: An Interprofessional Health Mentors Programme,” Clinical Teacher 11, no. 4 (2014): 301–306. 24917101 10.1111/tct.12222

[8] A. Trefzer , J. T. Jackson , K. McKee , and K. Dellinger , “Introduction: The Global South and/in the Global North: Interdisciplinary Investigations,” Global South 8, no. 2 (2014): 1–15.

[9] H. Spencer‐Oatey , Culturally Speaking Second Edition: Culture, Communication and Politeness Theory (Bloomsbury Publishing, 2008).

[10] J. E. Pierce , “Culture: A Collection of Fuzzy Sets,” Human Organization 36, no. 2 (1977): 197–200.

[11] J. M. Frambach and M. A. Martimianakis , “The Discomfort of an Educator's Critical Conscience: The Case of Problem‐Based Learning and Other Global Industries in Medical Education,” Perspectives on Medical Education 6 (2017): 1–4. 28050880 10.1007/s40037-016-0325-x PMC5285285

[12] S. D. Brookfield , Teaching for Critical Thinking Tools and Techniques to Help Students Question Their Assumptions (CA Jossey‐Bass.—References—Scientific Research Publishing [Internet], 2012) [cited 2024 Sep 2]. Available from: https://www.scirp.org/reference/referencespapers?referenceid=1054386.

[13] D. A. Sims , When I Say … Global South and Global North, vol. 58 (Medical Education. John Wiley and Sons Inc., 2024), 286–287. 10.1111/medu.15263 37963543

[14] C. Harber , Education and International Development: Theory, Practice and Issues (Symposium Books Ltd., 2014).

[15] T. Naidu , “Southern Exposure: Levelling the Northern Tilt in Global Medical and Medical Humanities Education,” Advances in Health Sciences Education 26, no. 2 (2021): 739–752, 10.1007/s10459-020-09976-9. 32500281

[16] G. Hofstede , “Dimensionalizing Cultures: The Hofstede Model in Context,” Online Readings in Psychology and Culture 2, no. 1 (2011): 12–2011, 10.9707/2307-0919.1014.

[17] A. Younas , S. Ramani , H. E. Popeijus , and M. Govaerts , “Learning From and With Patients: The Role of Culture,” Journal of CME 12, no. 1 (2023): 2259757, 10.1080/28338073.2023.2259757. 37795129 PMC10547442

[18] E. Paternotte , S. van Dulmen , N. van der Lee , A. J. J. A. Scherpbier , and F. Scheele , “Factors Influencing Intercultural Doctor‐Patient Communication: A Realist Review,” Patient Education and Counseling 98, no. 4 (2015): 420–445. 25535014 10.1016/j.pec.2014.11.018

[19] C. Taylan and L. T. Weber , ““Don't Let Me Be Misunderstood”: Communication With Patients From a Different Cultural Background,” Pediatric Nephrology 38 (2023): 643–649, 10.1007/s00467-022-05573-7. 35930048 PMC9842546

[20] D. L. Alden , J. Friend , P. Y. Lee , et al., “Who Decides: Me or We? Family Involvement in Medical Decision Making in Eastern and Western Countries,” Medical Decision Making 38, no. 1 (2017): 14–25, 10.1177/0272989X17715628. 28691551

[21] D. Hunter , J. McCallum , and D. Howes , “Defining Exploratory‐Descriptive Qualitative (EDQ) Research and Considering Its Application to Healthcare,” Journal of Nursing Health Care 4, no. 1 (2019): 14–25.

[22] L. Varpio , E. Paradis , S. Uijtdehaage , and M. Young , “The Distinctions Between Theory, Theoretical Framework, and Conceptual Framework,” Academic Medicine 95, no. 7 (2020): 989–994. 31725464 10.1097/ACM.0000000000003075

[23] N. Emmel , “Purposeful Sampling,” in Sampling and Choosing Cases in Qualitative Research: A Realist Approach (SAGE Publications Ltd, 2013), 33–45.

[24] H. Kallio , A. M. Pietilä , M. Johnson , and M. Kangasniemi , “Systematic Methodological Review: Developing a Framework for a Qualitative Semi‐Structured Interview Guide,” Journal of Advanced Nursing 72, no. 12 (2016): 2954–2965. 27221824 10.1111/jan.13031

[25] P. Rowland , K. R. MacKinnon , and N. McNaughton , “Patient Involvement in Medical Education: To What Problem Is Engagement the Solution?” in Medical Education (John Wiley & Sons, Ltd, 2021), 37–44, 10.1111/medu.14200. 32350875

[26] R. Khalife , M. Gupta , C. Gonsalves , et al., “Patient Involvement in Assessment of Post‐Graduate Medical Learners: A Scoping Review,” Medical Education 56 (2022): 602–613, 10.1111/medu.14726. 34981565

[27] V. Braun and V. Clarke , “Using Thematic Analysis in Psychology,” Qualitative Research in Psychology 3, no. 2 (2006): 77–101, 10.1191/1478088706qp063oa.

[28] G. Hofstede . “The 6 Dimensions Model of National Culture by Geert Hofstede [Internet],” Geert Hofstede. 2021 [cited 2023 Nov 17]. Available from: https://geerthofstede.com/culture‐geert‐hofstede‐gert‐jan‐hofstede/6d‐model‐of‐national‐culture/.

[29] R. L. Nadeau , Asian Religions: A Cultural Perspective (John Wiley & Sons, 2014).

[30] G. Hofstede , “Dimensionalizing Cultures: The Hofstede Model in Context,” Online Readings in Psychology and Culture 2, no. 1 (2011): 919–2307.

[31] H. L. Adam , C. M. Giroux , K. Eady , and K. A. Moreau , “A Qualitative Study of Patients' and Caregivers' Perspectives on Educating Healthcare Providers,” Canadian Medical Education Journal 12, no. 4 (2021): 7–16. 34567301 10.36834/cmej.71541 PMC8463222

[32] H. Meng‐Jie and G. Chi , “The Hierarchical Structure of Chinese Higher Education System,” US‐China Education Review 5, no. 12 (2015): 825–830.

[33] J. Massé , S. Grignon , L. Vigneault , G. Olivier‐D'Avignon , and M. C. Tremblay , “Patients' Perspectives on Their Motivations for Participating in Non‐Clinical Medical Teaching and What They Gain From Their Experience: A Qualitative Study Informed by Critical Theory,” Advances in Health Sciences Education 29, no. 1 (2024): 217–243. 37382856 10.1007/s10459-023-10262-7 PMC10927881

[34] E. Kangasjarvi , J. Forsey , J. S. Simpson , and S. L. Ng , ““We're Back in Control of the Story and We're Not Letting Anyone Take That Away From Us”: Patient Teacher Programs as Means for Patient Emancipation,” Advances in Health Sciences Education 29, no. 2 (2024): 487–505. 37455294 10.1007/s10459-023-10255-6

[35] N. Cvetanovska , R. L. Jessup , A. W. Shee , S. Rogers , and A. Beauchamp , “Patients' Perspectives of Factors Influencing Active Participation in Healthcare Interactions: A Qualitative Study,” Patient Education and Counseling 114 (2023): 107808. 37263050 10.1016/j.pec.2023.107808

[36] S. Desai , F. Chen , S. Reddy , and A. McLaughlin , “Measuring Women's Empowerment in the Global South,” Annual Review of Sociology 48, no. 1 (2022): 507–527.

