# Supplementary material for: Active Patient Involvement in Healthcare Education in the Global South: Perspectives From Pakistan
Source: Clin Teach. 2025 Aug 26;22(5):e70184. doi: 10.1111/tct.70184 (PMC12380654; doi:10.1111/tct.70184)
Supplement: Supplementary file 1 — Data S1: tct70184‐sup‐0001‐Supplementary_File.docx. Supporting Information. [file TCT-22-e70184-s001.docx]

**Supplementary File A: Interview Guide**

| **Section 1: Patients' Perceptions of Good Performance**   1. Can you describe your experience(s) of receiving treatment or consultation from a dental student?   Follow-up prompts:   - - What, for you is nice and appropriate?   - How would you describe a student who provided good care?   - Can you give examples of behaviors that stood out for you? - In what areas in which a student could improve or do better as they performed in your consultation?   Follow up prompts:   - When would you feel less well treated? - Can you provide an example of a situation where a student could have done better?     **Section 2: Patients' Involvement in Feedback & Assessment**  We are trying to make sure that students get patients feedback, because we feel that this is the best feedback for them to improve. In the future, after we design our program to improve student performance would you be willing to ….  3. Play a role or be involved in dental education / training of students who want to become a dentist?  Follow up prompts:   - How do you think you could fulfil this role? - Would you feel comfortable in fulfilling this role? - What do you think students might learn from this interaction?   4. How would you feel if you, as a patient, were asked to give feedback to the student about their performance?  Follow-up prompts:   - - Do you feel this is a valuable thing for you to do?   - Why do you feel it is important that you do this?   - Is there anything you would be concerned about, when providing feedback?   5. Which aspect of a student’s performance do you think you could provide feedback to students on?  Follow-up prompts   - Are there any specific areas where you feel patients can offer useful insights? - Do you have any further thoughts, feelings, hesitations, etc. to providing feedback   6. How do perceive your self-playing a role in the education of these students ?  Follow-up prompts:   - - - What would motivate you to be involved?     - What aspects of student training do you think patients should be part of?     **Section 3: Patients’ Preferences for Feedback Provision**  7. In what ways would you be willing to provide this feedback?  Follow-up Prompts:   - Why do you feel that this method of providing feedback would be more convenient for patients? - Why do you feel this method of providing feedback would work best for you? - Are there any challenges you foresee in providing feedback this way?     **Section 4: Factors Influencing Patients’ Perspectives on Assessment**    8. How would you feel if you, as a patient, were asked to play a formal role as an assessor and judge a student’s performance, e.g. by providing a score or mark  Follow-up Prompts:   - - What are your thoughts on patients playing this role?   - Are there any concerns or reservations you would have?   Is there anything else you’d like to share? |
| --- |
